# Fast, Automated Implementation of Temporally Precise Blind Deconvolution of Multiphasic Excitatory Postsynaptic Currents

**DOI:** 10.1371/journal.pone.0038198

**Published:** 2012-06-26

**Authors:** Daniel Andor-Ardó, Erica C. Keen, A. J. Hudspeth, Marcelo O. Magnasco

**Affiliations:** 1 Howard Hughes Medical Institute and Laboratory of Sensory Neuroscience, The Rockefeller University, New York, New York, United States of America; 2 Laboratory of Mathematical Physics, The Rockefeller University, New York, New York, United States of America; Mount Sinai School of Medicine, United States of America

## Abstract

Records of excitatory postsynaptic currents (EPSCs) are often complex, with overlapping signals that display a large range of amplitudes. Statistical analysis of the kinetics and amplitudes of such complex EPSCs is nonetheless essential to the understanding of transmitter release. We therefore developed a maximum-likelihood blind deconvolution algorithm to detect exocytotic events in complex EPSC records. The algorithm is capable of characterizing the kinetics of the prototypical EPSC as well as delineating individual release events at higher temporal resolution than other extant methods. The approach also accommodates data with low signal-to-noise ratios and those with substantial overlaps between events. We demonstrated the algorithm’s efficacy on paired whole-cell electrode recordings and synthetic data of high complexity. Using the algorithm to align EPSCs, we characterized their kinetics in a parameter-free way. Combining this approach with maximum-entropy deconvolution, we were able to identify independent release events in complex records at a temporal resolution of less than 250 µs. We determined that the increase in total postsynaptic current associated with depolarization of the presynaptic cell stems primarily from an increase in the rate of EPSCs rather than an increase in their amplitude. Finally, we found that fluctuations owing to postsynaptic receptor kinetics and experimental noise, as well as the model dependence of the deconvolution process, explain our inability to observe quantized peaks in histograms of EPSC amplitudes from physiological recordings.

## Introduction

The deconvolution tasks involved in astronomy, fluorescence microscopy, and electrophysiology display several useful parallels (Table 1). Although the nomenclature and dimensionality are different in these three cases, the mathematical model that describes the system as a convolution of a signal with an impulse-response function is essentially the same. Reconstructing the location and intensity of each object from the superposition of blurred images is known as *deconvolution*. Sometimes the basis of the blurring is known theoretically; in an ideal, diffraction-limited telescope, for example, it is an Airy function. An algorithm is said to be *blind* if, in addition to reconstructing the positions of the objects, it also reconstructs the impulse-response function associated with the blurring. An important subclass of such problems is characterized by a data set that is largely empty, peppered with only very few objects or events. Such problems are called *sparse* because one may safely assume that the information in the data is captured concisely by the locations and intensities of a small number of spatially point-like or temporally brief objects. Sparseness of the original signal introduces important mathematical simplifications that make the reconstruction process more tractable. As a result, reconstruction of sparse signals by deconvolution has been applied to many different fields. We present here a deconvolution algorithm that aids in the analysis of complex but sparse electrophysiological recordings obtained in the course of studying synaptic transmission.

**Table 1:** Analogous components in three different inverse problems.

	Electrophysiology	Astronomy	Fluorescence microscopy
*Signal*	Neurotransmitter release accompanying vesicle fusion	Position of a star	Location of a fluorescence source
*Filter*	Shape of excitatory postsynaptic current (EPSC)	Point-spread function of telescope	Point-spread function of imaging system
*Data*	Current trace	Photograph	Z-stack of images
*Dimensionality*	One	Two	Three

**Figure 1:**
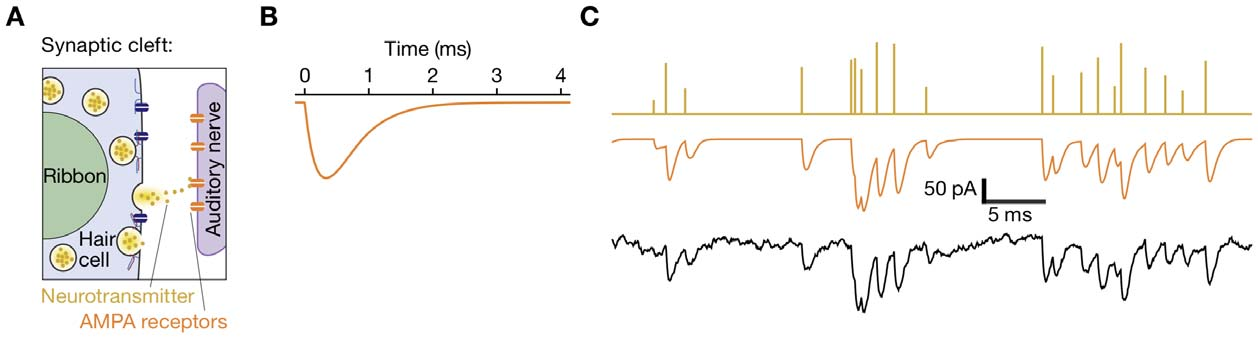
Structure and responses of a hair cell’s ribbon synapse. **A**, Fast exocytosis owing to the fusion of a synaptic vesicle with the presynaptic cell’s plasma membrane floods the synaptic cleft with neurotransmitter. Postsynaptic receptors, in particular AMPA receptors at the hair-cell synapse, open and allow the flow of current into the postsynaptic nerve terminal on the right. **B**, The impulse response of the system is the excitatory postsynaptic current (EPSC) of stereotyped amplitude and timecourse evoked by the release of transmitter from a single presynaptic vesicle. Postsynaptic AMPA receptor channels open and close stochastically; here the mean current is shown for 150 channels. Although the channels open quickly, the risetime of the measured current is limited by the time constant of the postsynaptic membrane. The reclosure of AMPA receptors is a slower process that is well approximated by a single exponential with a time constant of 1–2 ms. **C**, Postsynaptic-current recordings are modeled statistically in three steps. In the first row, peaks in the density of neurotransmitter in the synaptic cleft are caused by stochastic fusions of vesicles in several size classes. In the second row, these spikes of neurotransmitter produce in the postsynaptic cell bursts of current shaped by the kinetics of receptor activation and deactivation (panel **B**). In the third row, noise from processes in the cell and the experimental apparatus are added to the postsynaptic signal. The purpose of our algorithm is to reconstruct the first record from observation of the third record, inferring and using the response shown in panel **B**.

Chemical synaptic transmission, which involves the exocytotic release of neurotransmitter from synaptic vesicles, results in excitatory postsynaptic currents (EPSCs) that can be measured electrophysiologically in a postsynaptic neuron by tight-seal, whole-cell recording. Records of such postsynaptic currents are often complex, with overlapping signals that display a large range of amplitudes (Fig. 1; [1]). Because this complexity results from the superposition of neurotransmitter release events from various ensembles of vesicles, the postsynaptic response to a single presynaptic release event, the unitary EPSC, provides an appropriate basis for characterizing a sparse representation. In the auditory system, an additional complication arises from the tendency of presynaptic release events to synchronize and therefore produce apparently unitary EPSCs that may in fact reflect responses to neurotransmitter from several presynaptic vesicles.

Although statistical analysis of the kinetics and amplitudes of complex EPSCs is essential in the development of mechanistic models of neurotransmitter release, multiphasic waveforms containing multiple overlapping EPSCs pose a challenge for existing algorithms. We therefore approached EPSC deconvolution using a Bayesian framework, constructing and then solving a generative model. We aimed to improve upon existing algorithms in several ways. First, we sought to distinguish individual events even as the temporal proximity of neighboring events approached the risetime of the EPSC. Second, variability in experimental conditions demanded that the algorithm adapt automatically to each recording situation without the need for the user to supply precise EPSC profiles. Finally, we desired a blind algorithm that could estimate the shape of the unitary EPSC directly from the EPSCs detected in each recording. We expected that obtaining a good estimate of the unitary EPSC’s shape–the impulse-response function–would result in increased temporal acuity of the algorithm, permitting an improved analysis of multiphasic waveforms. We developed and implemented such an algorithm, then evaluated its performance on real and synthetic data.

## Methods

The algorithm was tested on data obtained from paired whole-cell patch-clamp recordings performed at hair-cell synapses in the amphibian papilla of the American bullfrog (*Rana catesbeiana*) as previously described [1].

## Results

We describe a general framework for a blind deconvolution algorithm, in which the observed data **x** are the sum of the signal **s** filtered through a convolution with impulse response **f** and noise **n**:
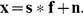
(1)


Given observation **x**, we wish to infer the signal and impulse response. We can write the posterior using Bayes’ rule:
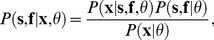
(2)in which 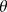 represents the remaining parameters such as the noise variance and regularization constant described below. Because we wish to minimize the number of parameters that must be provided to the algorithm by the user, we intend to automatically estimate or marginalize over several of them.

The likelihood 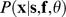 describes the character of the noise **n**, which for the noise in electrophysiological experiments is typically additive and Gaussian. Although **s** and **f** appear symmetrically in the likelihood, they have different properties and priors and therefore require different approaches to their numerical solution. These differences and constraints are captured by the prior 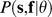. The signal is sparse, meaning that **s** is zero over most of its length. At any time when **s** is non-zero an event may have occurred; these abrupt spike-like events are spread along the length of **s** (Fig. 1C). In contrast, **f** is dense, much shorter than **s**, and mostly smooth (Fig. 1B). Lastly, we know that our signal must be everywhere positive and the impulse response must be everywhere negative. This problem is ill-posed because the number of degrees of freedom in the variables we wish to infer, **s** and **f**, exceeds that of the data **x**. There is an infinity of ways of maximizing the likelihood by fitting the noise, so a regularizer is needed to guide the algorithm toward the type of sparse solution that we require.

A specific aim of our algorithm is to analyze electrophysiological records with complex EPSCs. We therefore developed a method that takes EPSC kinetics into account to accurately disentangle most complex EPSCs. Fitting complex EPSCs by multiple events cannot be achieved by simply extending the process used to fit a single event: the nature of the task changes dramatically when events are allowed to overlap. For instance, although fitting a single Gaussian to data is trivial, fitting a sum of two or more Gaussians is challenging and is an active area of research in the field of clustering [2]. A special case of deconvolution occurs when the likelihood is separable in the data, all events being separated by a time greater than the length of the filter. The equations then simplify substantially and each event can be fitted individually (Appendix S1). But unless the data are separable in the sense described here–that is, containing no complex EPSCs–an algorithm must overcome the challenge of complex EPSCs by optimizing the signal globally rather than on an event-by-event basis.

A consequence of our sparseness prior is a non-linear filtering process, described below as the deconvolution step, which we use to separate **s** from **f**. Linear deconvolution filters, such as the Wiener filter, do not break the symmetry between **s** and **f**, and hence algorithms that use linear filters cannot be blind but require **f** to be fully specified *a priori* [3]–[5].

With the prior and likelihood defined, the posterior must be characterized. A point estimate, in the form of the maximum or mean of the posterior, is a common and convenient way to convey information about the posterior, and is also practical from the point of view of an experimentalist seeking an optimal way to visualize the signal. However, it is difficult to solve for signal and filter simultaneously. Although Monte Carlo techniques are applicable to the problem, we opted to make use of approximation techniques that are nearly as accurate but considerably faster. With this approach, the run-time of the algorithm scales approximately linearly with the length of the data.

We decompose the problem into two parts. During the first step, deconvolution, we find the optimal signal given the data and a preliminary estimate of the filter. During the second step, filter estimation, we improve our estimate of the filter given the data and our current estimate of the signal. We alternate between these two steps in a way analogous to expectation maximization.

### Deconvolution

Starting with the data **x** and a filter **f**, we find a sparse representation by optimizing the strength of a maximum-entropy regularizer. A regularizer is a cost function introduced into the optimization procedure to ensure the smoothness, magnitude, or some other property of the representation being sought, and often corresponds from the Bayesian perspective to the logarithm of a prior. A common family of regularizers seeks to minimize the *L_p_* norm of the signal vector **s**: 
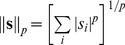
. There are efficient optimization techniques for deconvolution under certain conditions, for instance when the regularizer is an *L*_1_ norm [6] or *L*_2_ norm [7]. The *L*_1_ norm ensures sparseness; neither enforces positivity of the signal. Although our algorithm works with a variety of priors, for instance a Gaussian or a Burg entropy, we primarily use the so-called quantified maximum entropy (QME) prior [8] defined by the negative of the entropy, the negentropy *H*:
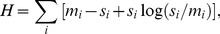
(3)in which 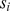 are the elements of **s**, and 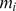 are the elements of the default signal **m**, usually set to the same constant value for all times. *H* has useful regularization properties by enforcing both positivity and sparsity in accordance with our stated prior assumptions about **s**. QME approximates the true maximum entropy prior while remaining numerically favorable due to the differentiability and convexity of *H*. In the following paragraph we outline the treatment of the QME prior implemented in the commercial software package MemSys5 [9].

When the filter is kept constant, the posterior is given by
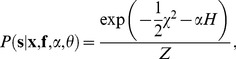
(4)in which 
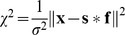
 is the sum of squared errors. We have introduced the regularization strength 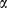, which determines the influence of the regularizer. The normalization constant or evidence, 
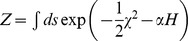
, which depends on **f**, 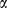 and additional parameters 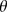, ensures that the posterior is a normalized probability density. Finding the best value of the regularization parameter is frequently a challenge in deconvolution problems, as sparsity comes about only at the correct value of 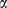. When 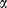 is too large, the prior dominates and the inferred signal lacks the full resolution available from the data. When 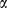 is too small, **x** corresponds to fitting the noise. Because of our Bayesian formulation, we can use the evidence framework [10] to optimize 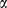. Instead of fully marginalizing over 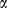, we maximize the evidence, *Z*, with respect to 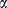, because fluctuations in 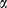 around its optimal value are very small. At the maximum, 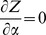 defines the optimal regularization 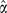. Due to the form of 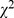, both the noise strength 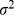 and the regularization constant 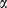 may be optimized at the same time, drawing attention to the fundamental importance of the signal-to-noise ratio in determining the resolution.

The point estimate of the signal is found by maximizing the posterior probability 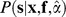 with respect to **s**, for 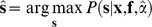. The presence of noise and the approximate nature of the prior means that 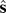 is nearly, but not exactly, zero in regions where there are no events but there is noise. Any point estimate suffers from the presence of spurious tiny events in 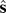; if we were to inspect the full posterior, we would find that their location and presence is highly uncertain. The process of obtaining a sparse representation by thresholding 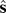 reduces the degrees of freedom significantly, typically by three orders of magnitude on our experimental data. This sparse representation is the key contribution of this first step, the maximum entropy deconvolution, and allows us to proceed to refine our estimate of **f**.

### Estimation of the Impulse Response

The quality of the estimate 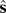 computed in the previous step depends crucially on the estimate of **f** we supplied. In this section, we show how to recursively improve the estimate of **f** over the initial guess.

We have formulated the problem such that the impulse response–the fundamental EPSC shape–is invariant, although the amplitude and location in time of each event differs. Conditional on 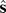, we estimate the impulse response non-parametrically by 
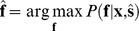
, in close analogy to 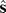.

Because **f** has finite support, its degrees of freedom are far fewer than provided by **x** and 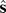. In this well-posed situation, if we consider the likelihood on its own, 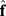 is the solution to the Wiener-Hopf equations [7]:
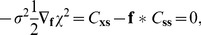
(5)in which *C* **_xs_** and *C* **_ss_** are cross-correlation functions defined by 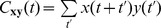 and * is the convolution operator.

We may simply use the sparse 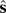 we inferred in the previous step and optimize **f**, taking care to enforce the negativity of **f** as we do so. When the events are all well-separated, this process is similar to identifying the isolated events and finding the impulse response that best fits them in a self-consistent manner.

### Convergence of Iteration

Successive applications of the deconvolution and impulse-response estimation steps quickly converge to the optimal signal and impulse response. Our computational approach is described in Appendix S1, with the nested nature of the resulting iterative loops shown in Figure S2. Despite the availability of a sparse representation following the second step above, the overlap among events implies that directly optimizing the amplitudes and times of events could converge to the wrong result. In general, it may also be prudent to run the algorithm from a variety of starting conditions in order to verify that the phenomenon under study is not strongly dependent on artifacts that have been introduced during deconvolution. When we explored different initial conditions with our data sets, we found that the algorithm always converged to the same solutions.

Unlike typical software packages, our method is defined not by an algorithm but by the generative model in the likelihood and the priors over the variables of interest. We can take advantage of additional capabilities by analyzing the posterior; we can, for instance, generate the error of an estimate. As better approximation techniques improve the quality of the solutions, the limit set by the signal-to-noise ratio may be approached to arbitrary proximity.

Although many aspects of EPSC deconvolution are analogous to image deconvolution, there are several features that are unique to electrophysiology. The additive noise in experimental recordings is often non-white and records can exhibit baseline drift over time. Furthermore, the impulse response is always negative. Because the AMPA receptors that generate EPSCs are fluctuating–unlike most point-spread functions–the impulse response is nondeterministic. The impulse response may vary owing to the presence of more than one synapse. Parameters of the model whose values we assume to be constant for the duration of a record, such as the power spectrum of the additive noise and the membrane time constant involved in the recording, may in fact exhibit non-stationarity. Incorporating the effects of AMPA receptors, multiple synapses, and non-stationarity would be a useful goal of future research.

### Performance on Physiological Data

**Figure 2:**
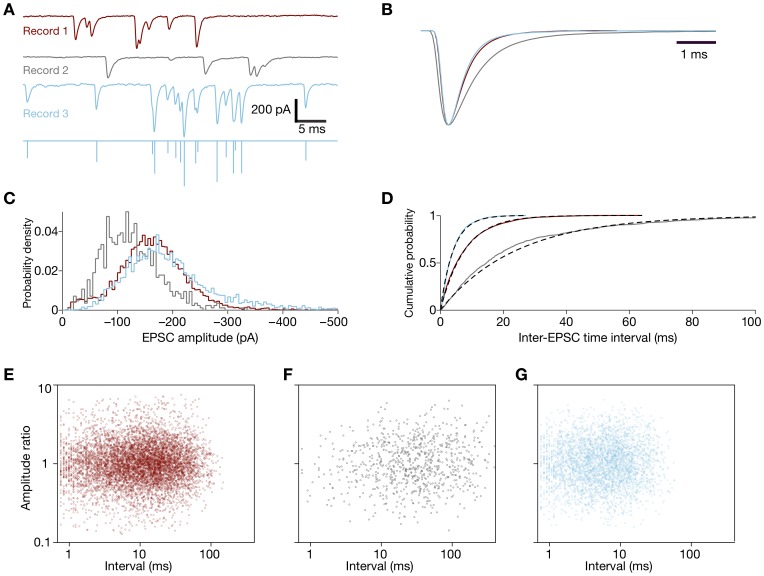
Processing of EPSC records. **A**, Selections from three records of postsynaptic current display typical EPSCs. The bottom trace shows the deconvolution of the third record. The color coding of the records applies as well to the subsequent panels. **B**, Estimates of the EPSC impulse response for the three experimental records of which segments are shown in panel **A** reveal the variability in EPSC kinetics. For records 1, 2 and 3, we detected respectively 7799, 825, and 3800 EPSCs. **C**, The probability distributions display the EPSC amplitudes detected by the algorithm for the three records. **D**, Cumulative probability distributions of inter-EPSC intervals for the three records (continuous lines) are adequately fit by single exponential functions (dotted lines). Perhaps because of a lack of true stationarity of the process, the magenta curve deviates most from a single exponential. **E–G**, Scatter plots relate the time delay to the amplitude ratio of successive pairs of EPSCs for the three records. No significant correlation between these variables is apparent.

To test the performance of our algorithm, we analyzed data recorded from the bullfrog’s amphibian papilla, an auditory end-organ whose spontaneous exocytosis reveals complex, highly overlapping EPSCs [1]. The experiments were conducted under protocols approved by the Institutional Animal Care and Use Committee of The Rockefeller University. In a first example, we sought to demonstrate the ability of our algorithm to identify individual EPSCs from complex records. From long, stable records (Fig. 2A) the algorithm readily recovered the characteristic sum-of-exponentials shapes of the constituent EPSCs (Fig. 2B). The distributions of EPSC amplitudes (Fig. 2C) were compatible with distributions found elsewhere [11]. The distributions of inter-EPSC time intervals were well approximated by exponential functions (Fig. 2D). We also characterized the data by examining correlations between event amplitudes and timing. Little or no correlation was apparent between amplitudes and interevent intervals in our recordings (Fig. 2E–G).

**Figure 3:**
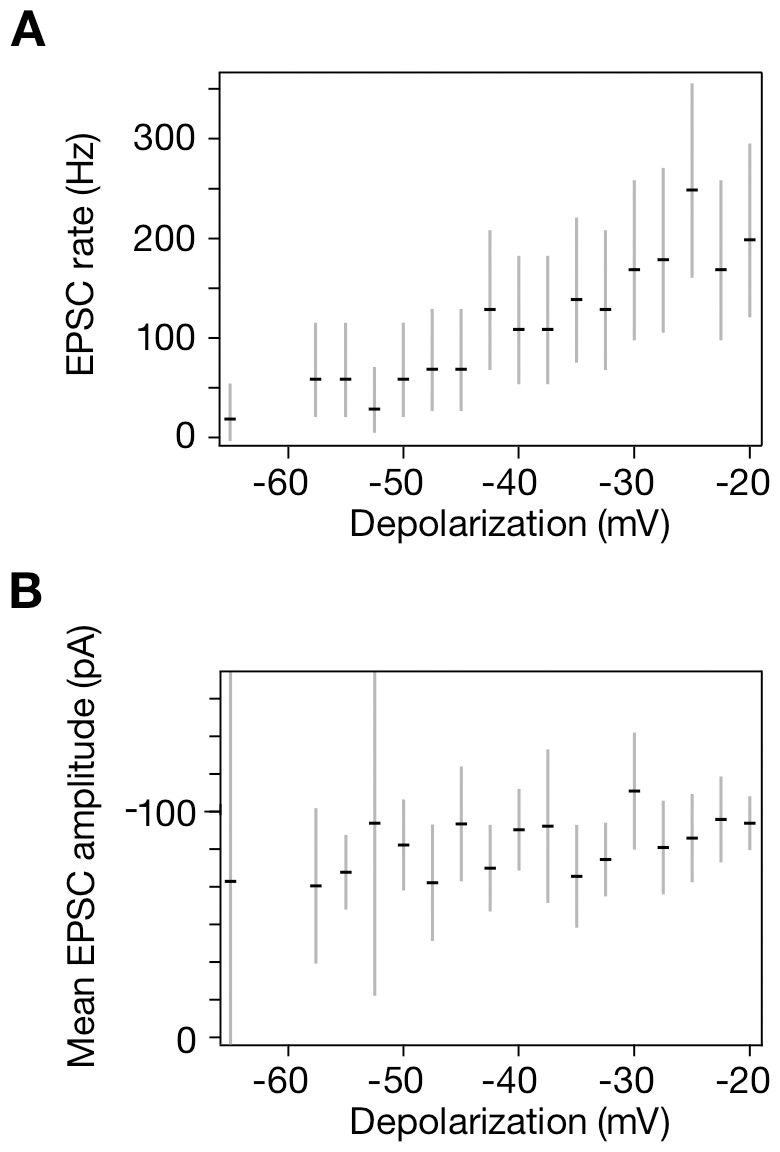
A test of the basis for the increased magnitude of EPSCs with progressive depolarization of the presyanptic hair cell. **A**, The mean EPSC rate grows appreciably as a function of depolarization. **B**, The mean EPSC amplitude displays negligible dependence on the extent of depolarization. The error bars indicate 95% confidence intervals. The measured mean amplitude of about −100 pA is consistent with that reported in Figure 4C of [11].

In a second example, we sought to use our algorithm to answer a physiologically important question: are stimulus-dependent increases in the postsynaptic current the consequence of an increase in the rate of EPSCs or an increase in the mean amplitude of EPSCs? Without deconvolving the signal, it is not possible to answer this question. We explored the complex waveforms resulting from an experiment in which the presynaptic cell was given a series of depolarizing steps between −65 mV and −20 mV. This procedure was a rigorous test of our algorithm, for large depolarizations produce postsynaptic currents that are highly complex and overlapping. Using our algorithm, we conclude that the increase in total postsynaptic current stems primarily from an increase in the rate of EPSCs (Fig. 3A) rather than an increase in their amplitudes (Fig. 3B).

### Performance on Synthetic Data

We also estimated the performance of our algorithm by analyzing synthetic data whose EPSC amplitudes, timings, shapes, and noise spectrum were fully controlled. We tested our algorithm’s ability to extract from a complex record the characteristic EPSC impulse response as well as the timing and amplitude of individual events. We also informally compared the performance of our algorithm to that of MiniAnalysis [12], a popular software program for the analysis of physiological recordings. MiniAnalysis represents a heuristic approach that provides flexibility at the cost of requiring operator assistance. By contrast, our global deconvolution approach is self-consistent and allows the program to run with less intervention.

Synthetic data were constructed by simulating AMPA receptors to generate EPSCs: at an instant drawn from a Poisson process, a fraction 0.1–0.5 of 150 receptors was selected to be open instantaneously by drawing from a binomial distribution. The receptors were allowed to reclose stochastically with a timescale of about 1 ms. The result was low-pass filtered with a time constant of approximately 0.25 ms and sampled at 50 µs intervals, parameters selected to match the membrane time constants and sampling frequencies of typical recordings, such as those in Figure 2. Additive Gaussian noise was filtered with a low-pass filter fitted to the shape of a typical noisy experiment and adjusted to a root-mean-square amplitude of 10 pA. The significance of coloring the noise is that the typical noise correlation time was about 1 ms, close in timescale to the typical time constant of EPSC decay, making the assay more challenging. For each test, the algorithm was run to recover the EPSC impulse response as well as the times and amplitudes of events.

**Figure 4:**
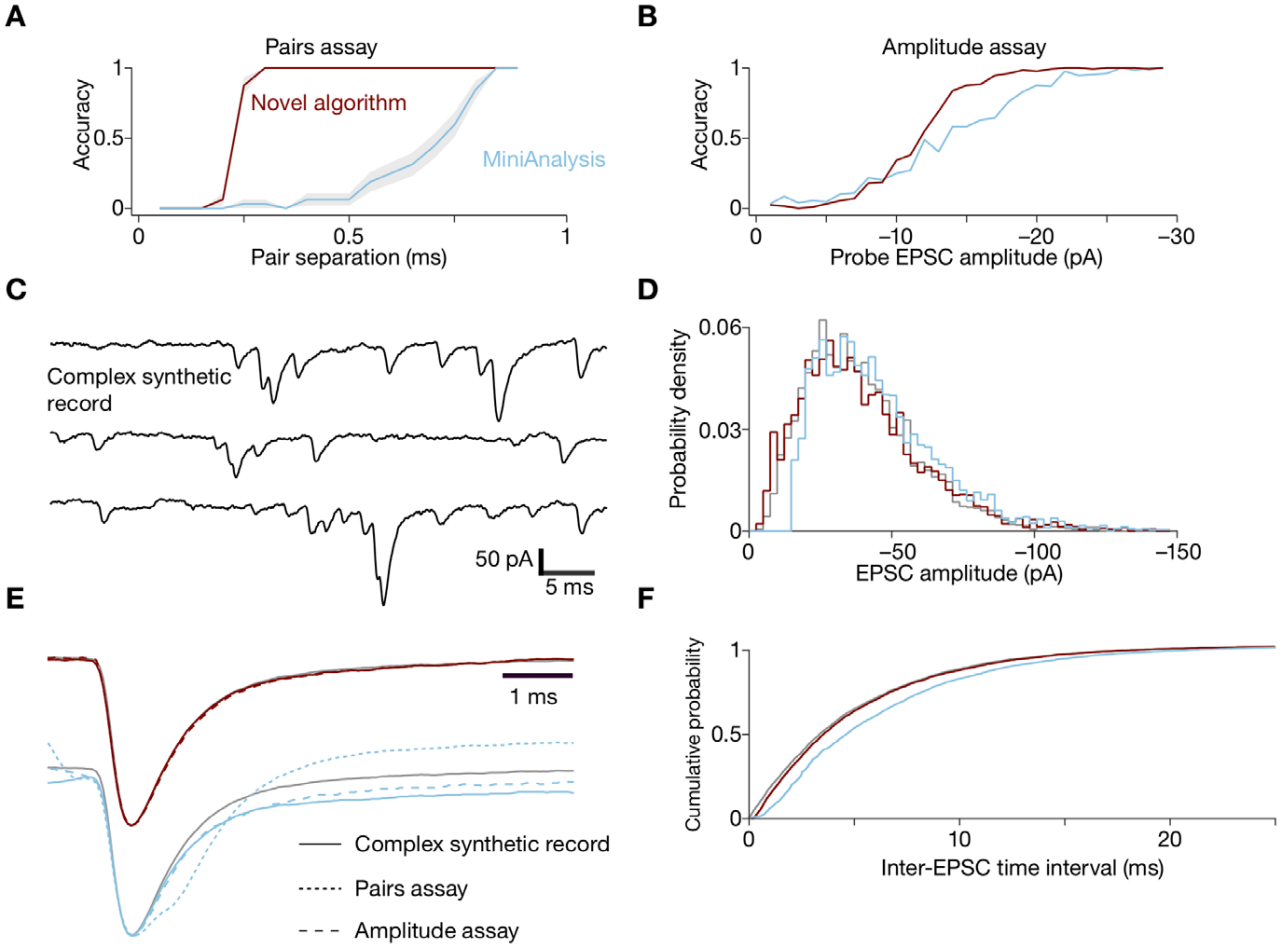
Evaluation of the algorithm’s performance. **A**, The novel algorithm reliably detects pairs of unitary EPSCs separated by about 0.25 ms, an interval about one-third that required by the MiniAnalysis program. In this and the subsequent panels, the results from the new algorithm are displayed in red and those from MiniAnalysis in blue. **B**, The accuracy of amplitude estimation is slightly improved by the new algorithm. **C**, A synthetic record consisting of 3968 EPSCs, of which three small segments are displayed, was used to evaluate the two procedures. **D**, The probability distribution of amplitudes determined by the new algorithm agreed more closely with the true distribution than did the result from MiniAnalysis. **E**, EPSC impulse-response functions were determined by the algorithms for the three schemas shown in this figure and compared to the true impulse response. “Pairs assay” and “Amplitude assay” refer to the tests plotted in panels **A** and **B**; “Complex record” refers to the test plotted in panel **C**. The new algorithm provided an excellent fit by all criteria. **F**, The new algorithm fit the true cumulative probability distribution of the inter-EPSC intervals, which was an exponential function.

To determine the temporal resolution of our algorithm, we tested its ability to detect individual EPSCs embedded in a series of paired EPSCs separated by increasing time delays. The separation at which individual events can be reliably distinguished depends on the signal-to-noise ratio, the event kinetics and amplitudes, and the algorithm. In this test paradigm, we achieved accuracy–that is, the fraction of test cases classified correctly–greater than 0.8 when EPSCs were separated by 250 µs (Fig. 4A). In contrast, EPSCs must be separated by at least 800 µs to obtain a similar accuracy with MiniAnalysis (Fig. S1).

To assess the amplitude sensitivity of our algorithm, we generated a series of individual EPSCs with amplitudes ranging from –1 pA to –30 pA (Fig. 4B). As with the temporal-resolution assay, the accuracy curves of the amplitude-sensitivity assay have a sigmoidal shape. Because EPSCs are non-overlapping in this assay, we were not analyzing a complex record. Nevertheless, our algorithm improved upon MiniAnalysis by achieving an accuracy of 0.8 at –14 pA; MiniAnalysis reached the same level of accuracy at –19 pA. The standard deviation of the error in amplitudes was 5 pA for our algorithm and 7 pA for MiniAnalysis. The jitter in timing estimates had a standard deviation of 50 µs for our algorithm and of 90 µs for MiniAnalysis.

To simulate a more realistic scenario, we synthesized complex records containing EPSCs with amplitudes drawn from a gamma distribution and intervals drawn from an exponential distribution (Fig. 4C). We compared the algorithm’s estimate with the true distributions of amplitudes and intervals (Fig. 4D and F).

For each of the assays, we asked the algorithm to retrieve the EPSC impulse-response function. We compared the results to the ideal function obtained by low-pass filtered, mean AMPA kinetics sampled at 50 µs intervals. MiniAnalysis experienced difficulties in aligning complex EPSCs and performed well only when well-separated EPSCs were present in the amplitude sensitivity assay (Fig. 4E). In contrast, our algorithm retrieved the correct impulse-response function after only one or two iterations.

### Detection of Multiquantal Peaks

**Figure 5:**
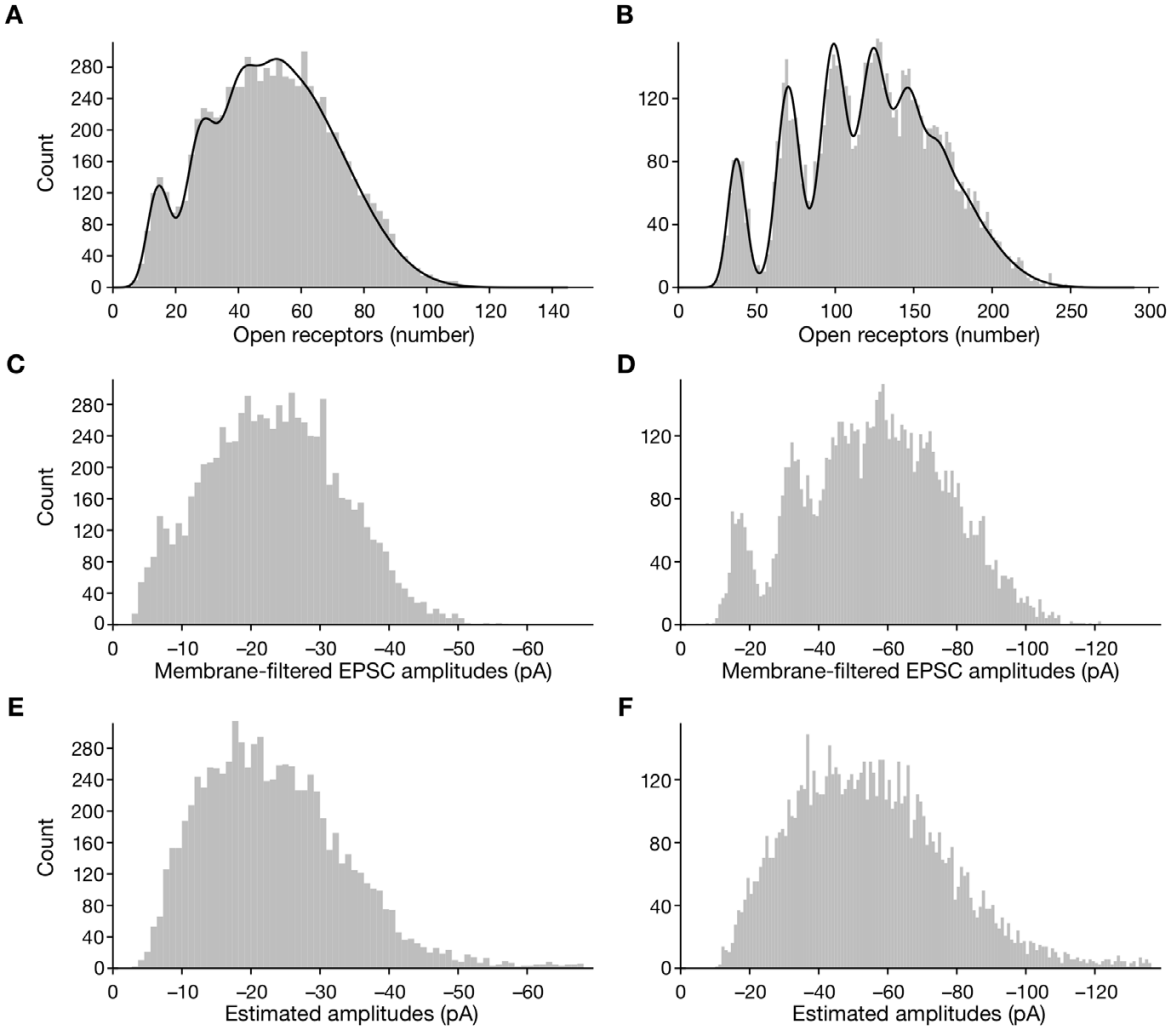
Fits of amplitude distributions for model data from simulations. **A**, The distribution of channel openings was derived for 8000 events with a model comprising 150 AMPA receptors. The magnitude of a single quantum was set to 0.1 of total receptor saturation, or 15 channels; the mean number of quanta per fusion event was 4 and the root-mean-square experimental noise was 10 pA. **B**, The distribution was derived from 8000 simulated events for a model with 300 AMPA receptors. The single quantum was set to 0.125 of total receptor saturation, or 37.5 channels. **C**, **D**, Convolving the data in respectively panels A and B with the kinetics of channel reclosure and the membrane time constant blurs both distributions. **E**, **F**, The final amplitude distributions, which include the additional effect of additive noise, lack distinct peaks.

Amplitude histograms of measured EPSCs are often used in electrophysiological studies to investigate the quantization of neurotransmitter release. To assess whether multiquantal events could be discerned with our algorithm, we performed simulations in which we fixed the quantities of neurotransmitter to be integer multiples of a smaller quantum and then calculated amplitude distributions at three stages between ligand binding and deconvolution. Stage one (Fig. 5A for a total of 150 receptors and Fig. 5B for 300 receptors) records the number of AMPA receptors open due to a burst of neurotransmitter. Stage two (Fig. 5C and D) includes filtering of the incoming current by the membrane time constant and the stochastic reclosure of AMPA receptors. Stage three (Fig. 5E and F) includes colored noise and subsequent deconvolution by our algorithm.

The blurring evident in the simulated records is a result of several sources of noise: stochastic ligand binding, stochastic channel reclosure, and additive noise. Additional noise may arise at stage one because neurotransmitter released from presynaptic vesicles is imperfectly quantized. Stages two and three may be contaminated by signals originating from different presynaptic cells or active zones, whose waveforms may be filtered to different extents before reaching the recording electrode.

The blurring of the EPSC amplitude distribution may be mitigated in at least two ways. First, increasing the number of AMPA receptors or the proportion of AMPA receptors that a single vesicle’s neurotransmitter opens will reduce the relative fluctuations in ligand binding and channel reclosure. Second, reducing the experimental noise will permit more accurate amplitude estimation by any deconvolution algorithm.

If the simple model used here captures a minimum of the stochastic processes involved in generating EPSCs, we may conclude that intrinsic fluctuations provide a rationale for the inability to observe quantization peaks in EPSC amplitude histograms derived from complex physiological recordings.

## Discussion

We have developed an automated self-consistent deconvolution algorithm for the analysis of challenging EPSC data. The statistical model on which the algorithm is based is simply stated and cleanly separated from the details of the approximations used in the implementation. Several numerical strategies allow the algorithm to run efficiently, requiring a temporal duration approximately proportional to the problem size. For instance, analyzing a 10 s recording sampled at 50 µs intervals (size 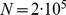
_)_ requires less than one minute on a mid-range workstation. We have demonstrated the algorithm’s applicability by deconvolving complex postsynaptic recordings from an auditory synapse. The analysis reveals that increasing presynaptic depolarization leads to more frequent postsynaptic events, rather than larger ones. A series of tests using synthetic data shows that the algorithm performs favorably compared to the widely used MiniAnalysis program.

Most algorithms require the noise level or the impulse response to be supplied by the user. Moreover, in algorithms that use a regularizer, for instance those based on Wiener filtering, the regularization constant must be adjusted empirically. Our algorithm automates the estimation of the impulse response, the noise, and the regularization constants. For users requiring manual control, however, these automatic steps may be selectively overridden. A switch changes the prior from QME to Gaussian, making the algorithm perform as a Wiener filter. The noise and regularization constants can be overridden and the impulse response need not be estimated from the data if it is known from previous experimentation.

Integral to the study of synaptic dynamics through EPSC measurements is the kinetics of the EPSCs themselves. A fundamental challenge facing all algorithms is that of separating multiple overlapping EPSCs from individual EPSCs that simply have intrinsic variability in their kinetics. Highly sparse records seem to indicate that EPSC kinetics do not vary wildly, so it is parsimonious to introduce that assumption into our analysis and classify multiphasic EPSCs as being due to multiple overlapping EPSCs. The possibility cannot be excluded, however, that when multiple vesicles fuse in synchrony there are additional factors modulating the postsynaptic response and thus changing the EPSC kinetics. It remains unlikely that the analysis of postsynaptic records alone will adequately resolve this issue.

The range of observed EPSC amplitudes and multiquantal release might emerge from a number of mechanisms, including synchronized exocytosis [13] and sequential fusion. A further possibility is prefusion, in which several vesicles combine to form a supervesicle prior to exocytosis. If the area of vesicle membrane is conserved during fusion, and if exchangers bring the concentration of neurotransmitter to some constant level, then a supervesicle originating from the fusion of *q* single vesicles experiences a volume increase–and hence an increase in transmitter content–from *q*-fold to *q* ^3/2^–fold that of a single vesicle. This change prior to or during the exocytotic process could further impair our ability to observe multimodal amplitude histograms.

Recent experiments have determined that a single presynaptic vesicle contributes only about a quarter to a half of the average postsynaptic EPSC amplitude at the hair cell’s auditory synapse [11]. Experimentally measured amplitude distributions nevertheless show little evidence of multiquantal peaks (Fig. 2C, [11]). Should we be able to find evidence of multiquantal release in EPSC amplitude distributions? We used the simulation framework developed here to investigate the distinct contributions of the intrinsic noise of AMPA receptor binding and kinetics, experimental noise, and the deconvolution process to the blurring of peaks in the distribution of EPSC amplitudes. For typical parameters of postsynaptic AMPA receptors, we found that multiquantal peaks are sufficiently blurred to obscure the evidence for multiquantal release. Stochastic ligand binding and channel reclosure usually suffice to make the amplitude distribution appear unimodal. Because we are unlikely to observe multiquantal peaks in such experimental amplitude histograms, they provide little evidence to test hypotheses of multiquantal neurotransmitter release.

We have developed a flexible framework to perform deconvolution of time series data in an efficient and automated manner. Any data in which underlying time-sensitive or rhythmic information has been blurred in a systematic way benefit from deconvolution. The applicability of this approach ranges from electrical current to gene-expression profiles and population fluctuations. We have explicitly demonstrated the algorithm’s relevance to experimental records containing stereotyped responses of varying amplitudes, such as those obtained during the recording of postsynaptic currents reflecting neuronal activity. Users can determine the statistics of events, such as their location and amplitude, or the shapes of events, even if the events are highly overlapping and the signal-to-noise ratio is low. By publishing the algorithm and its source code in an open manner at https://github.com/andorardo/fade, we encourage users to add our algorithm to their repertory.

## Supporting Information

Figure S1 Detection parameters used for analysis with MiniAnalysis.
(TIFF)

Figure S2 Schematic of the algorithm’s nested loops.
(PDF)

Appendix S1 The implementation and computational aspects of the algorithm are described in the Appendix.
(DOC)
