# Supplementary figures and images for: Fast, Automated Implementation of Temporally Precise Blind Deconvolution of Multiphasic Excitatory Postsynaptic Currents
Source: PLoS One. 2012 Jun 26;7(6):e38198. doi: 10.1371/journal.pone.0038198 (PMC3383690; doi:10.1371/journal.pone.0038198)

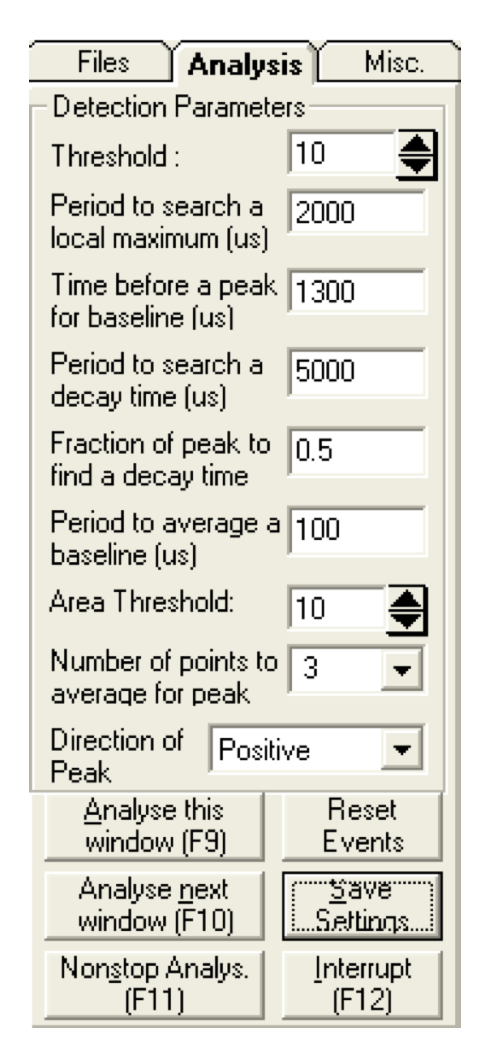

Supplement: Figure S1 — Detection parameters used for analysis with MiniAnalysis. (TIFF) [file pone.0038198.s001.tiff]
