# Supplementary material for: Fast, Automated Implementation of Temporally Precise Blind Deconvolution of Multiphasic Excitatory Postsynaptic Currents
Source: PLoS One. 2012 Jun 26;7(6):e38198. doi: 10.1371/journal.pone.0038198 (PMC3383690; doi:10.1371/journal.pone.0038198)
Supplement: Figure S2 — Schematic of the algorithm’s nested loops. (PDF) [file pone.0038198.s002.pdf]

Maximize $P(\mathbf{x} \mid \alpha)$

$\mathbf{s} \Leftarrow \mathbf{s} - \mathbf{J}^{-1}\mathbf{g}$

Find $\mathbf{J}^{-1}\mathbf{g}$

Search directions $\mathbf{B}^{n}\mathbf{g}$

Until $\Delta\mathbf{s}$ is small

Use $\mathrm{trace}(\mathbf{M}) \approx \mathbf{r}^{T}\mathbf{M}\mathbf{r}$
