# Supplementary material for: Fast, Automated Implementation of Temporally Precise Blind Deconvolution of Multiphasic Excitatory Postsynaptic Currents
Source: PLoS One. 2012 Jun 26;7(6):e38198. doi: 10.1371/journal.pone.0038198 (PMC3383690; doi:10.1371/journal.pone.0038198)
Supplement: Appendix S1 — The implementation and computational aspects of the algorithm are described in the Appendix. (DOC) [file pone.0038198.s003.doc]

# Supporting Information

# Fast, automated implementation of temporally precise blind deconvolution of multiphasic excitatory postsynaptic currents

Daniel Andor-Ardó^1,2*^, Erica C. Keen^1^, A. J. Hudspeth^1^, and Marcelo O. Magnasco^2^

**1** Howard Hughes Medical Institute and Laboratory of Sensory Neuroscience, The Rockefeller University, New York, New York, United States of America, **2** Laboratory of Mathematical Physics, The Rockefeller University, New York, New York, United States of America * Email: daa@cantab.net

### Appendix

### Implementation

Both the deconvolution and impulse response-estimation steps involve a series of optimization loops. The size of the data vector **x** is *N*, which can be 10^5^ or larger. Our main computational challenge is therefore to perform all computations without having to explicitly store or operate on any *N*‑by‑*N* matrices. Because the spatial resolution of the signal needs not coincide with the sampling rate of the data, the quantization of **s** can differ from that of **x.** However, obtaining high-resolution estimates is one of our goals, so we will not initially reduce the resolution of the signal below that of the data. Matching the lengths of **x** and **s** also offers computational advantages.

Noise in an electrophysiological recording is often colored and may contain electronic noise with sharp peaks in the power spectrum. We can construct a whitening filter and apply it to the data. Because the whitening filter distorts the parts of the data corresponding to impulse response as well, we must apply the whitening filter to **f**. The commutativity of convolution allows us to do this.

##### Deconvolution step

The outermost loop of the deconvolution step leads to the optimization of $\alpha$ and implicitly $s^{2}$. We start with a large value of $\alpha$, far from the optimum, a situation in which the posterior is dominated by the prior and we accordingly know its maximum; for instance, $s=m$ in the case of the QME prior and $s=0$ in the case of the Gaussian prior of a Wiener filter. We intend to reduce $\alpha$ to the value that maximizes the evidence *Z*. To do so, we perform a line search in $\text{ln}\alpha$ space to find the root . We save the values of ln *Z* so we can estimate the accumulation of evidence through approximation by quadrature of the integral

. (S1)

The optimization of $\alpha$ is analogous to the gradual reduction of temperature in the technique of simulated annealing.

For a given value of $\alpha$ we calculate the maximum *a posteriori* estimate of **s** by Newton-Raphson iteration. This is equivalent to maximizing the log posterior or minimizing the regularized sum of errors or free energy, , with respect to **s**. As we approach the solution to , we terminate the iterations when the fluctuations in **g** become smaller than that expected due to the noise level [1]. For Newton-Raphson iteration to converge it is important to start near the solution, hence the large initial value of $\alpha$ and small steps in $\text{ln}\alpha$ are essential.

The evidence *Z* is an integral over **s** with respect to a measure set by the invariant volume element [**h**]d**s**, in which [**h**] is the Jacobian. It is equivalently the Hessian of *H* and is typically diagonal as indicated by the brackets [1,2]. Only in the case of a Gaussian prior is the evidence integral also Gaussian; otherwise we make a Laplace approximation about . Even in the Gaussian approximation the integral involves the determinant of a vast *N*‑by‑*N* matrix. Fortunately, we need only estimate the gradient , which depends on the trace rather than the determinant and is easier to estimate.

Each of the Newton-Raphson steps, the termination criterion, and the evidence integral require the application of the inverse of the Hessian of *G*. This is computationally the most expensive operation in the loop. We can express the Hessian as , in which and **K** is the Hessian of $\frac{1}{2}c^{2}$. We can calculate the effect of application of all the matrices without having to store their elements. For instance, the application of **K** to vector **s** is given by ; that is, the convolution of the signal with the autocorrelation of the filter divided by $s^{2}$. With the problem in this form, there exist sophisticated Krylov-space techniques to solve the linear problem $(A+\alpha I)y=b$ for vector **y** in the space spanned by basis vectors . The maximal number of basis vectors required, *n*+1, which gives the termination criterion, is also calculated [1,3]. These techniques work well when **A** is sparse, a property determined by the support of the impulse response, *L*, relative to the data length, *N*. In the case for EPSC recordings, **A** is predictably sparse. A record of length $N=10^{6}$ typically requires the storage and application of basis vectors. Careful implementation of the algorithm regains *O*(*N*) algorithmic complexity when .

It remains only to estimate the trace of a number of matrices. The trace is given by the expectation , in which the random vectors **r** are delta correlated: . For large *N* the trace is well approximated by using only a few random vectors **r**.

The relationship between the nested loops of the algorithm is illustrated schematically in Figure S2.

##### Impulse response-estimation step

Because **f** has small support of length *L*, the Wiener-Hopf equations (Eqn. 5) can be cast in *L*‑by‑*L* matrix form. The optimization of **f** can then be done by constrained minimization, bringing to the problem our physiological knowledge that the EPSC corresponds to current flowing in only one direction. This method, essentially a form of reverse correlation, works for all forms of **s**.

Equation 5 has a particularly simple form when events are widely spaced, such that . We describe this case for completeness. The Wiener-Hopf equations reduce to

(S2)

which can be solved iteratively for the set of times of the events {*t_i_*}, the set of amplitudes of the events {*s_i_*}, and the impulse-response function *f*(*t*). The last of the three equations raises our intuition about how events are combined optimally to form an estimate of the impulse-response function: because larger events have a relatively greater signal-to-noise ratio, events should be averaged with weighting proportional to their respective amplitudes.

### References

1 Skilling J (1993) Bayesian numerical analysis. In: Grandy J, Milonni P, editors. Physics and Probability. Cambridge University Press

2 Kass RE (1989) The geometry of asymptotic inference. Statist Sci 4: 188-234

3 Gibbs MN (1997) Bayesian Gaussian Processes for Regression and Classification. PhD thesis. Cambridge University
